# P21-activated kinase regulates oxygen-dependent migration of vascular endothelial cells in monolayers

**DOI:** 10.1080/19336918.2021.1978368

**Published:** 2021-09-22

**Authors:** Satomi Hirose, Yugo Tabata, Kazuki Sone, Naoyuki Takahashi, Daisuke Yoshino, Kenichi Funamoto

**Affiliations:** a Graduate School of Biomedical Engineering, Tohoku University, Aoba-ku, Sendai, MiyagiJapan; b Institute of Fluid Science, Tohoku University, Aoba-ku, Sendai, MiyagiJapan; c Institute of Engineering, Tokyo University of Agriculture and Technology, Koganei, TokyoJapan; d Graduate School of Engineering, Tohoku University, Aoba-ku, Sendai, MiyagiJapan

**Keywords:** Collective cell migration, hypoxia, vascular endothelial monolayer, microfluidic device, p21-activated kinase

## Abstract

The collective migration of vascular endothelial cells plays important roles in homeostasis and angiogenesis. Oxygen tension *in vivo* is a key factor affecting the cellular dynamics. We previously reported hypoxic conditions promote the internalization of vascular endothelial (VE)-cadherin and increase the collective migration of vascular endothelial cells. However, the mechanism through which cells regulate collective migration as affected by oxygen tension is not fully understood. Here, we investigated oxygen-dependent collective migration, focusing on intracellular protein p21-activated kinase (PAK) and hypoxia-inducing factor (HIF)-1α. The results indicate that the oxygen-dependent variation of the migration speed of vascular endothelial cells is mediated by the regulation of VE-cadherin through the PAK pathway, as well as other mechanisms via HIF-1α, especially under extreme hypoxic conditions.

## Introduction

Cells migrate by controlling cell-substrate and cell-cell adherent molecules through intracellular signaling and remodeling the cytoskeleton [1,2]. In addition to the dependence of single-cell migration on cell-extracellular matrix (ECM) interactions, a group of cells collectively migrates while communicating signals via cell-cell junctions mechanically and chemically [3]. Cells in a confluent state migrate while exerting forces on each other and forming multiple clusters [4]. Such collective cell migration behavior is different from single-cell migrations, showing parallel motions, swirling motions, and extrusion in each cell cluster [5,6]. Collective cell migration plays important roles in the development of tissues and organs and the maintenance of homeostasis [7–9]. However, variations of collective cell migration due to environmental factors are not fully understood. Cellular migratory behaviors are changed by intracellular signal transduction responding to mechanical and chemical stimuli [10–12]. Oxygen tension surrounding cells is one factor that changes cellular dynamics including cell migration [13–15]. The *in vivo* oxygen tension is lower than the atmospheric one (21% O_2_). For example, physiological oxygen levels in peripheral tissues are approximately 4–7.5% O_2_ depending on the organ [16], and it can be further decreased to severe hypoxic levels by disease and inflammation [17–19]. Decreases in oxygen tension have been reported to cause angiogenesis by endothelial cells [14] and metastasis and proliferation of cancer cells [15]. *In vitro* studies, such as wound healing (scratch) assays, have shown that a hypoxic environment encourages the migration of endothelial cells [20] and cancer cells [21]. However, those studies have not characterized collective cell migration in a confluent monolayer, because only the migration of cells covering the scratched non-confluent part is observed. Recently, cellular experiments using microfluidic devices have allowed real-time observation of cultured cells with strictly controlled oxygen tension [22–24]. By using custom-made microfluidic devices [13,25], we found that a hypoxic exposure of ~2% O_2_ causes increases in permeability [26] and collective cell migration [27] in a vascular endothelial cell monolayer. These changes in endothelial behaviors and dynamics are caused in part by weakened cell adhesion as a result of hypoxia-induced internalization of vascular endothelial (VE)-cadherin, a cell-cell adhesion molecule.

Hypoxia-inducible factors (HIFs) are major proteins involved in the cellular response to hypoxic stress [28]. HIF-1α in particular regulates cell migration and contributes to its stabilization [29,30]. p21-activated kinase (PAK) promotes stabilization of HIF-1α through the generation of reactive oxygen species, and HIF-1α further activates PAK, acting as a positive feedback loop [31]. Therefore, we hypothesized that the hypoxic environment enhances cell migration via VE-cadherin internalization by HIF-1α stabilization and PAK activation. However, we lack knowledge about the mechanisms through which oxygen tension induces VE-cadherin internalization via the PAK-HIF-1α positive feedback loop. In addition, the relationship between the collective migration of vascular endothelial cells and oxygen tension remains unknown.

Here, in this article, we provide evidence that PAK is involved in the regulatory mechanisms of hypoxia-dependent cell migration. we investigated the oxygen-dependent collective migration of vascular endothelial cells and the underlying mechanisms involving the regulation of VE-cadherin, through the PAK pathway, and regulation by HIF-1α. Vascular endothelial monolayers were formed in microfluidic devices that can control the oxygen tension around cultured cells. The migratory behavior of cells was observed with time-lapse imaging and analyzed by particle image velocimetry (PIV) to measure migration velocity under various oxygen tensions. Localizations of HIF-1α and VE-cadherin in vascular endothelial cells after hypoxic exposure were quantified with immunofluorescence staining. In addition, the collective migration of vascular endothelial cells with PAK inhibition and HIF-1α stabilization by chemical reagents was measured to test the mechanisms of oxygen-dependent migratory behavior.

## Materials and methods

### Microfluidic device

**Figure 1.**
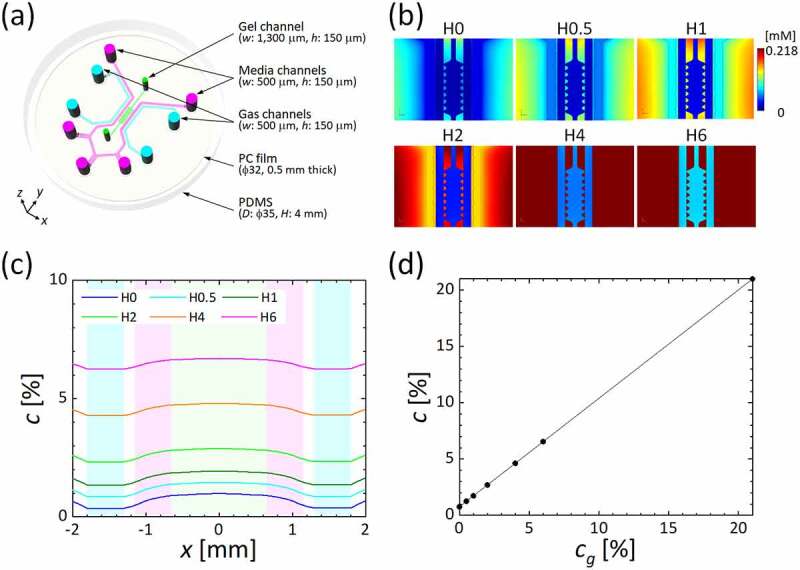
Microfluidic device. (a) Schematic of the device. (b) Oxygen concentration on the bottom glass coverslip. (c) Oxygen concentration profiles on the bottom glass coverslip across the media and gel channels. The green, pink, and blue shaded regions indicate the gel, media, and gas channels. (d) Relationship between oxygen concentration at the centerline of the media channel and that in the gas mixture supplied to both gas channels

Microfluidic devices with the ability to control oxygen tension, fabricated from poly(dimethylsiloxane) (PDMS), a polycarbonate (PC) film, and a glass coverslip, were used (Figure 1(a)) [32]. The device diameter and thickness are 35 mm and 4 mm, respectively. Inside the device, a central gel channel (1,300 µm wide) into which hydrogel is filled to mimic the ECM is flanked by a Y-shaped media channel (500 µm wide). On both sides of the media channels, a pair of gas channels (500 µm wide) to which gas mixtures at predefined oxygen concentrations are supplied is located to control oxygen tension inside the device. The media and gas channels are separated by 150-µm wide PDMS partitions. Cell culture media in the media channel and the gas mixtures supplied to the gas channels do not directly contact each other, and the oxygen tension in the device is controlled by gas exchange between the channels through the PDMS. The height of all channels is 150 µm. Additionally, a PC film (32 mm diameter and 0.5 mm thick) is embedded in the device at a distance of 0.5 mm above the bottom glass coverslip to prevent oxygen infusion from the atmosphere.

The controllability of oxygen concentration with the device was evaluated by three-dimensional numerical analysis using commercial finite element software (COMSOL Multiphysics ver. 5.5; COMSOL AB, Sweden) [13]. The gas mixtures supplied to the gas channels were assumed to be an incompressible fluid with a density of 1 × 10^3^ kg/m^3^ and a viscosity of 1 × 10^−3^ Pa∙s. The Navier-Stokes equations and the equation of continuity were solved for gas flow in the gas channels. For the boundary conditions of the gas flow, a constant flow rate of 18 ml/min was set at the inlet of each gas channel, and zero pressure was given at the outlet. A no-slip condition was applied on the channel wall. Then, the spatial distribution of oxygen concentration in the device was computed by solving the convection-diffusion equation. The device was assumed to be placed in an atmosphere containing 21% O_2_, and the initial conditions of oxygen concentration for PDMS, PC, and media were defined according to Henry’s equation. In addition, a partition condition of oxygen concentration was applied at the interface between PDMS and media and gel channels to consider the difference in oxygen solubility for each substance. A no-flux condition was applied on the bottom glass coverslip. Then, the oxygen concentration was computed for conditions in which a gas mixture containing 0–21% O_2_ was supplied to both gas channels. Hereafter, each condition is termed based on the oxygen concentration in the supplied gas mixture: the normoxic condition when oxygen concentration was 21% O_2_ is named ‘N’; hypoxic conditions when oxygen concentrations were below 21% O_2_ are noted with ‘H’ followed by the numerical value of the oxygen concentration in the gas mixture, i.e., H0–H6. The physical properties of the media, gas, gel, PDMS, and PC as set in the computational analysis are summarized in Table 1. The device was capable of generating a minimum uniform hypoxic condition of 0.8% O_2_ in the media channel when an anoxic gas mixture was supplied to the gas channels (H0) (Figure 1(b) and (c)). The oxygen concentrations generated at the centerline of the media channel by conditions H0, H0.5, H1, H2, H4, H6, and N were 0.8, 1.3, 1.7, 2.7, 4.6, 6.6, and 21% O_2_, respectively (Figure 1(d)). The deviation of the oxygen concentration in the media channel against that in the gas mixture supplied to the gas channels was assumed to be caused by oxygen infusion from the atmosphere into the device. Nevertheless, a clear linear relationship was confirmed between the values of oxygen concentration in the supplied gas mixture and in the media channel, implying that the device can control the oxygen concentration around cultured cells.

**Table 1.** Physical properties of each component and parameters for numerical simulations

	Medium	Gas	Gel	PDMS	PC film
Density, *ρ* [kg/m^3^]	1.0 × 10^3^	1	1.0 × 10^3^		
Viscosity, *μ* [Pas]	1.0 × 10^−3^	1.0 × 10^−5^			
Diffusivity of oxygen, *D* [m^2^/s]	2.0 × 10^−9^	2.0 × 10^−5^	2.0 × 10^−9^	4.0 × 10^−9^	2.0 × 10^−12^
Solubility of oxygen, *S* [mM/atm]	0.218		0.218	1.25	1.25
Péclet number, *Pe*		100			
Average velocity, *U* [m/s]		4			
Flow volume, *Q* [ml/min]		18			
Oxygen tension, *c* [%]	21	0–21			

The device was fabricated using a silicon wafer with the channel pattern laminated by photolithography. The channel pattern was transferred to the PDMS layer (0.5 mm thick) (Silgard 184 Silicone Elastomer Kit; The Dow Chemical Company, USA) composed of a 10:1 mixture of base and curing agent by soft lithography. A PC film with holes to access the inlets and outlets of the channels (3.2-mm diameter holes for the media and the gas channels, 1.6 mm holes for the gel channel) was placed on the top of the PDMS layer, and then additional fresh PDMS was poured over the PC film to 4 mm in height and cured in an oven (60°C) overnight. The cured PDMS was peeled off, cut to be 35 mm in diameter, and punched to create the inlets and outlets of the channels (2-mm diameter holes for the media and gas channels, 1 mm holes for the gel channel). After the PDMS mold and a glass coverslip (35 mm in diameter) were sterilized, they were bonded together after hydrophilization for 90 s using a plasma cleaner (PDC-32G; Harrick Plasma, USA). Immediately after that, a poly-D-lysine (PDL) solution of 1 mg/ml (P7886; Sigma-Aldrich, USA) was injected to the gel and media channels, and the device was placed in an incubator (5% CO_2_, 37°C) for at least 4 h to improve adhesion of the hydrogel to the channel wall. Then, the PDL solution was aspirated, and the channels were washed with sterile water twice and dried in the oven overnight to let the channel surface become hydrophobic. Type I collagen solution (354236; Corning, USA) prepared at 2.5 mg/ml and pH 7.4–7.5 was filled in the gel channel and polymerized in the incubator for 40 min. Cell culture medium (EGM-2, CC-3156; Lonza, Switzerland) was then injected into the media channel, and the device was incubated overnight for mechanical stabilization of the collagen gel. Moreover, the media channel was coated with Matrigel (356230; Corning) of 2.0 mg/ml on ice for better cell adhesion just before cell seeding.

### Cellular experiments

Human umbilical vein endothelial cells (HUVECs, C2519A; Lonza) cultured with EGM-2 medium at less than the ninth passage were used [26,27]. HUVECs cultured on a cell culture dish were harvested before they reached confluency by trypsin treatment (CC-5012 and CC-5002; Lonza). Then, a cell suspension at a density of 5 × 10^6^ cells/ml was prepared and injected into the media channel. By culturing the cells for three days and changing the EGM-2 medium every day, a vascular endothelial cell monolayer was formed covering the media channel.

Collective cell migration in the vascular endothelial monolayer of HUVECs was then observed under controlled oxygen tension. The device with the vascular endothelial cell monolayer was placed in a stage incubator (INUBSF-ZILCS; Tokai HIT, Japan) (5% CO_2_, 37°C) mounted on a microscope (EVOS FL Cell Imaging System; Life Technologies, USA). A humidified gas mixture containing 0, 0.5, 1, 2, 4, 6, or 21% O_2_ and 5% CO_2_, balanced with N_2_, was supplied to the both gas channels to generate uniform oxygen conditions in the device (conditions H0, H0.5, H1, H2, H4, H6, or N; see Figure 1(d)). After the gas mixture was supplied, phase-contrast microscopic images of the vascular endothelial cell monolayer on the bottom glass coverslip were obtained every 10 min for 5 h. The migration velocity of the HUVECs was measured by PIV with the time-series of the microscopic images. In the PIV analysis, JPIV open-source software [33] was utilized, and microscopic images obtained at every 30 min between 1 h and 5 h after the observation started were analyzed in order to eliminate the effects of device movement and temperature change observed just after the experiment started. A rectangular region of 512 × 960 pixels (440 × 825 µm) set in the media channel was divided into small regions of interest (ROIs) of 8 × 8 pixels (6.88 × 6.88 µm), and displacement of each ROI between the two sequential time points was measured by calculating the cross-correlation function. The ROI displacements were then converted to the migration velocity of the HUVECs.

In our previous studies [26,27], internalization of VE-cadherin caused by hypoxic exposure at 2–3% O_2_ loosened cell-cell adhesion, resulting in an increased velocity of collective migration of vascular endothelial cells. Following these previous results, in the present study we investigated the effects of hypoxic exposure at various oxygen concentrations and changes in cell-cell junctions by VE-cadherin on collective cell migration. Since the internalization of VE-cadherin is reportedly regulated by activation of PAK [34], cell-cell adhesion by VE-cadherin should be strengthened by the administration of a PAK inhibitor. Hence, the collective migration of HUVECs was observed after adding the PAK inhibitor FRAX597 (6029; Tocris Bioscience, UK). FRAX597 was dissolved in dimethyl sulfoxide (DMSO) (049–07213; FUJIFILM Wako Pure Chemical Corporation, Japan) at 1 mM and then added to EGM-2 medium at a final concentration of 100 nM. In addition, as a positive control for hypoxic conditions, the collective migration of HUVECs was also observed by adding cobalt(II) chloride (CoCl_2_) (232696; Sigma-Aldrich) to chemically simulate a hypoxic environment. CoCl_2_ was dissolved in distilled water at 25 mM, and then added to EGM-2 medium at final concentrations of 100, 200, or 300 µM. For experiments in which chemical reagents were added, the cell culture medium was changed to that containing each chemical one day before the experiment (the second day of cell culture in the device).

### Immunofluorescence staining

After 2 or 5 h of exposure of the vascular endothelial monolayer to each oxygen condition by supplying the gas mixture to the gas channels, the HUVECs were fixed with 4% paraformaldehyde phosphate buffer solution (163–20145; Wako Pure Chemical Industries, Japan) for 10 min and then permeabilized with 0.1% Triton X-100 (T9284; Sigma-Aldrich) in phosphate-buffered saline (PBS) (P5119; Sigma-Aldrich) for 5 min. The cells were then blocked with 1% Block Ace (DS Pharma Biomedical, Japan) in Dulbecco’s PBS (DPBS, D8537; Sigma-Aldrich) (BA-DPBS) for 30 min to prevent nonspecific absorption of the antibodies described below. HIF-1α in cells exposed to each oxygen condition for 2 h was evaluated, referring to our former study [27] that showed the maximum nuclear translocation of HIF-1α after 2 h of exposure to hypoxia. VE-cadherin in cells exposed to each oxygen condition was also evaluated at the same time as well as at the endpoint of the 5-h time-lapse observation of the cells. HIF-1α or VE-cadherin were labeled with individual mouse monoclonal antibodies (ab1; Abcam plc., UK; or sc-9989; Santa Cruz Biotechnology, USA) at dilutions of 1:100 in BA-DPBS for 1 h, followed by subsequent staining with fluorescence-labeled anti-mouse secondary antibody (A11001 or A11032; Thermo Fisher Scientific, USA) at a dilution of 1:100 in PBS for 1 h. Early endosome antigen 1 (EEA1) was labeled with a rabbit polyclonal antibody (ab2900; Abcam, USA) at dilution of 1:100 in BA-DPBS for 1 h, followed by subsequent staining with fluorescence-labeled anti-rabbit secondary antibody (A11008; Thermo Fisher Scientific) at dilution of 1:100 in PBS for 1 h. Cell nuclei were stained with DAPI (D21490; Thermo Fisher Scientific) at 1 μg/ml. The immunofluorescence staining was conducted at room temperature. Between the individual steps, the cells were washed twice with DPBS.

Microscopic images of HUVECs on the bottom of the media channel were acquired using a confocal laser scanning microscope (LSM800; Carl Zeiss Microscopy, Germany). Twenty images of the cells on the horizontal plane (*xy*-plane) were taken at 0.63 µm intervals in the vertical (*z*-axial) direction, and the maximum intensity of the images was projected to the *xy*-plane. The projected images were then analyzed using open-source software (ImageJ; National Institutes of Health, USA) to quantitatively evaluate hypoxic sensing by the cells and changes in cell-cell junctions. To evaluate cellular hypoxia sensing, the average fluorescence intensities of HIF-1α in the whole image and in cell nuclei, *Ī*_whole_ and *Ī*_nucleus_, were calculated, respectively, and the relative average intensity *Ī*_nucleus_/*Ī*_whole_ was then obtained as the ratio of nuclear translocation of HIF-1α [27]. For changes in cell-cell junctions, the areas surrounded by the outer and inner outlines of VE-cadherin, *A*_out_ and *A*_in_, were measured, respectively, and the ratio of the VE-cadherin area to the total cell area, A*_cad_ (= *A*_cad_/*A*_out_ = (*A*_out_-*A*_in_)/*A*_out_), was evaluated as an indicator for VE-cadherin internalization [26,35]. Additionally, VE-cadherin internalization through endocytosis was evaluated based on the colocalization of VE-cadherin and EEA1. The average fluorescence intensities of VE-cadherin colocalized with EEA1 and in the cytoplasm, *Ī*_endosome_ and *Ī*_cytoplasm_, were calculated, respectively, and the relative average intensity *Ī*_endosome_/*Ī*_cytoplasm_ was then obtained.

### Data and statistical analysis

The collective migration of HUVECs was measured using four microfluidic devices for cell culture media with/without FRAX597 and CoCl_2_ under each oxygen condition. Immunofluorescence observations of the cells were performed with four devices for each target protein and each oxygen condition. Microscopic images were obtained at five arbitrary locations for each microfluidic device, and 20 images in total were utilized for the image analysis. To evaluate VE-cadherin, three cells were randomly chosen in each microscopic image, and 60 cells in total were employed for quantitative evaluation. Significant differences between the results under different conditions of oxygen concentration and chemical addition were assessed by one-way analysis of variance (ANOVA) or two-way ANOVA followed by *post hoc* Tukey’s test. In each test, statistical significance was inferred at *P* < 0.05.

## Results

### Oxygen tension dependence of vascular endothelial cell migration

**Figure 2.**
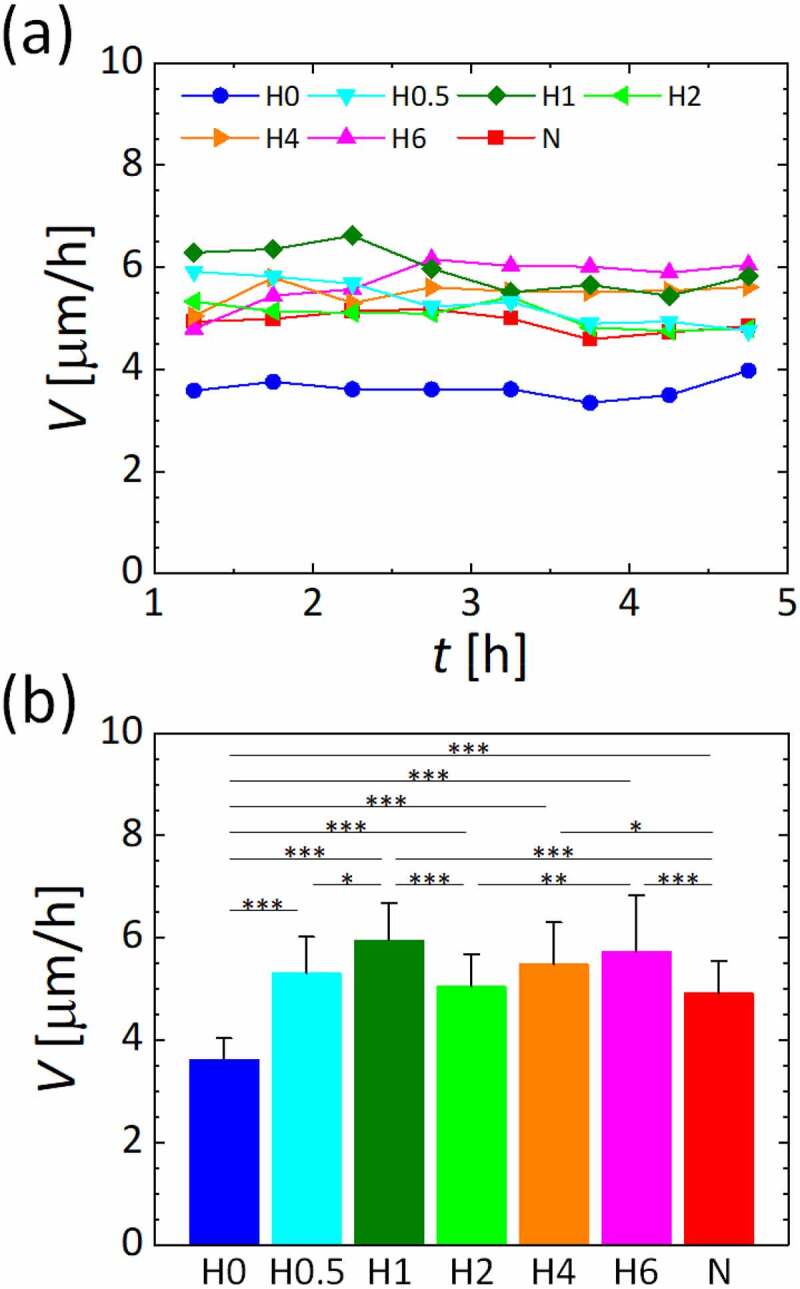
Migration speed of HUVECs in the monolayer under various oxygen conditions generated in the microfluidic device. (a) Time variations of spatially averaged values and (b) spatiotemporally average values during the measurement period between 1 h and 5 h after supplying gas mixtures into the gas channels. Error bars show standard deviation. Significant changes in the migration speed of the cells by oxygen concentration were assessed by one-way ANOVA followed by Tukey’s *post hoc* test for multiple comparisons. *p < 0.05; **p < 0.01; ***p < 0.001

We first measured the migration of HUVECs by PIV analysis based on the time-series of phase-contrast microscopic images (Fig. S1 and Movie S1). Variations of the spatially averaged migration speed every 30 min between 1 h and 5 h after supply of the gas mixtures were of an almost constant value for each oxygen condition (Figure 2(a) and S2). The spatiotemporally averaged migration speed was calculated by the spatially averaged migration speed over 4 h for each oxygen condition (Figure 2(b)). Compared to the normoxic condition N, the migration speed significantly increased under hypoxic oxygen conditions H1, H4, and H6, whereas it significantly decreased under oxygen condition H0. The oxygen conditions H0.5 and H2 did not contribute to changes in migration speed. A local maximum of the migration speed was observed under oxygen condition H1.

**Figure 3.**
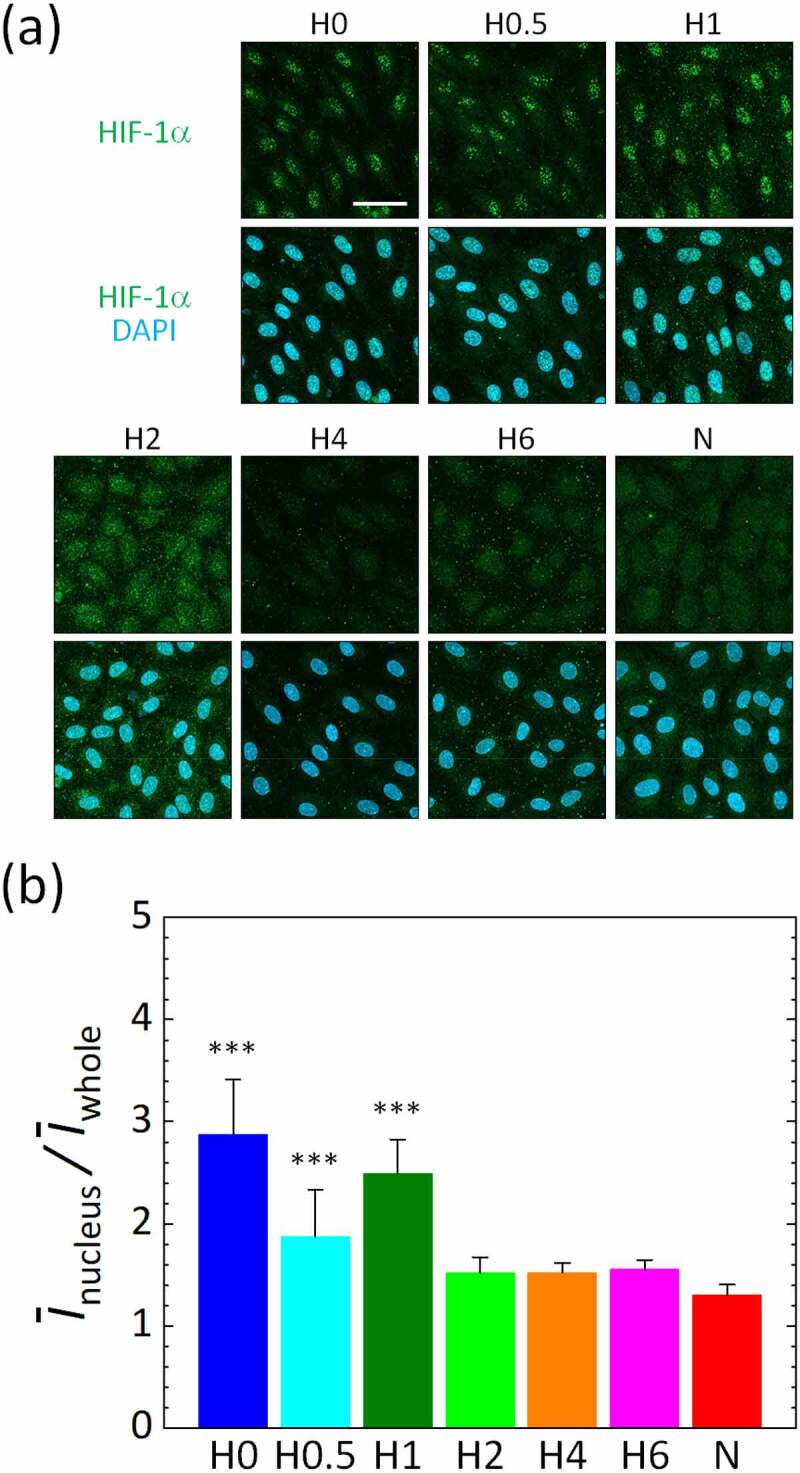
Nuclear translocation of HIF-1α in HUVECs after 2 h of exposure to various oxygen conditions generated in the microfluidic device. (a) Representative images of maximum intensity projections of confocal microscopic images of HUVECs to the *xy*-plane. Scale bar = 50 μm. (b) Average intensity of HIF-1α in the nucleus relative to that in the whole image, *Ī*_nucleus_/*Ī*_whole_. Error bars show standard deviation. Significant differences of the nuclear translocation of HIF-1α by oxygen concentration were assessed by one-way ANOVA followed by Tukey’s *post hoc* test for multiple comparisons. ***p < 0.001 vs. N

We then confirmed oxygen sensing in the cells on the basis of HIF-1α localized in the cell nucleus after 2 h of the gas mixture supply for each oxygen condition (Figure 3(a)). The ratio of the nuclear translocation of HIF-1α in HUVECs significantly increased under lower oxygen conditions H0, H0.5, and H1 compared to normoxic condition N, though no significant changes were observed in oxygen conditions H2, H4, and H6 (Figure 3(b)). The ratio of the nuclear translocation of HIF-1α was also at a local maximum value under condition H1, under which the maximum migration speed was also observed. The nuclear translocation of HIF-1α was maximized under the most severe hypoxic condition H0, where the migration speed significantly decreased compared to the normoxic condition N.

### Changes in migration and oxygen sensing of vascular endothelial cells by inhibition of PAK

**Figure 4.**
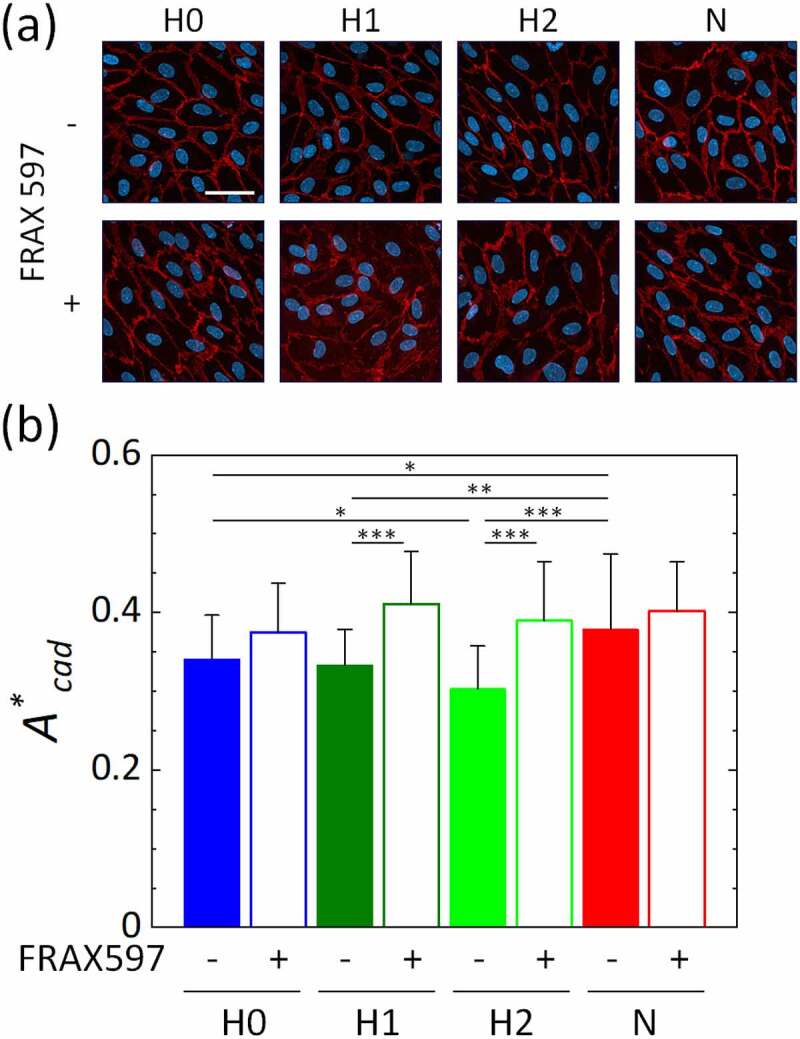
VE-cadherin of HUVECs, treated without/with the PAK inhibitor FRAX597 after 5 h of exposure to various oxygen conditions generated in the microfluidic device. (a) Representative images of maximum intensity projections of confocal microscopic images of HUVECs to the *xy*-plane. Scale bar = 50 μm. (b) Ratio A*_cad_ of the VE-cadherin area to the total cell area. The metric was quantified for 60 cells from four device under each condition. Error bars show standard deviation. Significant differences of the VE-cadherin area by oxygen concentration were assessed by two-way ANOVA followed by Tukey’s *post hoc* test for multiple comparisons. *p < 0.05; **p < 0.01; ***p < 0.001

We explored what derives the oxygen-dependent changes in cell migration. VE-cadherin internalization was found to promote cell migration under hypoxic conditions in our previous study [27]. Since PAK forms a positive feedback loop with HIF-1α and is known to regulate VE-cadherin, it could be related to cell migration under hypoxia. We thus examined the relationship between cell migration and PAK, focusing on hypoxic conditions H0, H1, and H2, and the normoxic condition N. With the supplementation of the PAK inhibitor FRAX597 to the cell culture medium, thick bands of VE-cadherin were observed around the cells under all oxygen conditions, implying that the cell-cell junctions were strong (Figure 4(a)). Here, the 2-h exposure of HUVECs to hypoxic conditions slightly increased the colocalization of EEA1 and VE-cadherin in the cytoplasm, implying VE-cadherin internalization through endocytosis (Fig. S3). Based on the quantified ratio A*_cad_ of the VE-cadherin area to the total cell area, PAK inhibition tended to stabilize VE-cadherin adherens junctions under all oxygen conditions (Figure 4(b)). In particular, significant stabilization of VE-cadherin junctions was observed under low oxygen conditions H1 and H2.

**Figure 5.**
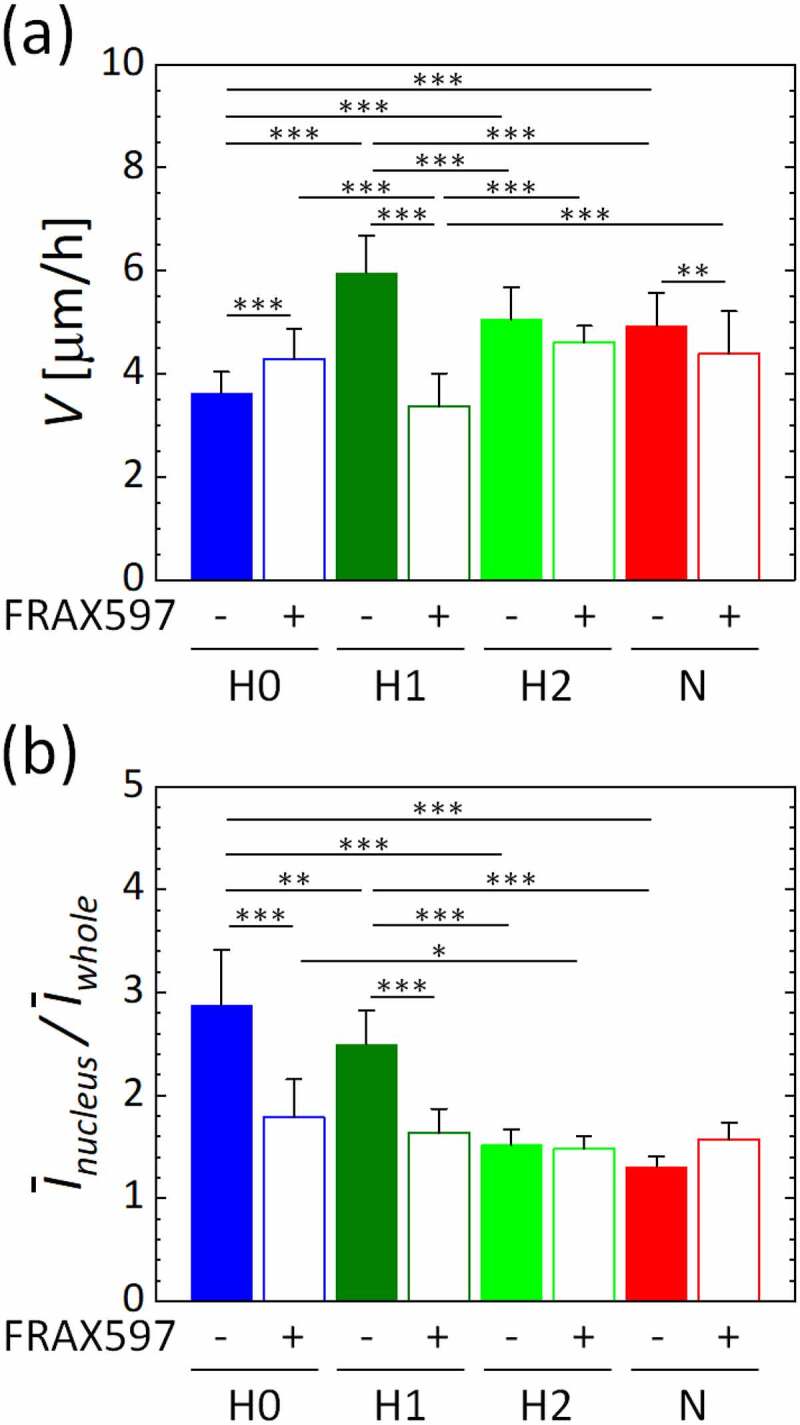
Inhibition of PAK in HUVECs by FRAX597. (a) The spatiotemporally averaged migration speed and (b) the average intensity of HIF-1α in the nucleus relative to that in the whole image, *Ī*_nucleus_/*Ī*_whole_. Error bars show standard deviation. Significant differences by oxygen concentration were assessed by two-way ANOVA followed by Tukey’s *post hoc* test for multiple comparisons. *p < 0.05; **p < 0.01; ***p < 0.001

We then measured the migration speed of HUVECs supplemented with FRAX597 between 1 h and 5 h after the start of gas supply (Figure 5(a) and S2). Under each oxygen condition, the spatially averaged migration speed was almost constant during the measurement period (Fig. S2(d)). PAK inhibition by FRAX597 significantly decreased the migration speed of HUVECs under oxygen condition H1, which corresponded to an enhanced VE-cadherin localization level due to the suppression of its internalization (Figure 4). In contrast, under the lowest oxygen condition H0, the migration speed increased despite the enhanced protein localization level of VE-cadherin by PAK inhibition. To further examine the relationship between the PAK and oxygen sensing, we evaluated the nuclear translocation of HIF-1α after 2 h of the gas mixture supply for each oxygen condition (Figure 5(b)). The nuclear translocation of HIF-1α was significantly decreased by PAK inhibition under oxygen conditions H0 and H1, showing almost the same values under all oxygen conditions. Vehicle DMSO treatment did not affect the experimental results of the migration speed or the stabilizations of HIF-1α and VE-cadherin (Fig. S4). These results imply that PAK is involved in the stabilizations of HIF-1α and VE-cadherin, which are related to the oxygen-dependent migration of HUVECs.

### Vascular endothelial cell migration under chemically-induced hypoxia

**Figure 6.**
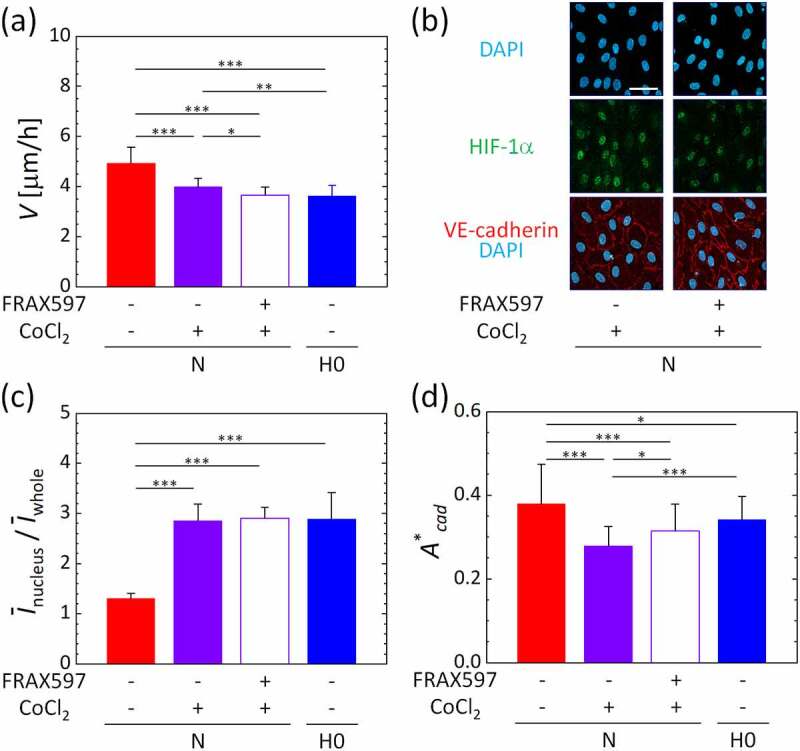
Inhibition of PAK in HUVECs by FRAX597 under chemically-induced hypoxia by 300 μM CoCl_2_. (a) The spatiotemporally averaged migration speed during the measurement period between 1 h and 5 h after supplying gas mixtures into the gas channels. (b) Representative images of maximum intensity projections of confocal microscopic images of HUVECs to the *xy*-plane. Scale bar = 50 μm. (c) Average intensity of HIF-1α in the nucleus relative to that in the whole image, *Ī*_nucleus_/*Ī*_whole_. (d) Ratio A*_cad_ of the VE-cadherin area to the total cell area. Error bars show standard deviation. Significant differences by oxygen concentration were assessed by one-way ANOVA followed by Tukey’s *post hoc* test for multiple comparisons. *p < 0.05; **p < 0.01; ***p < 0.001

To ascertain whether PAK directly regulates HIF-1α-mediated oxygen sensing in HUVECs, we investigated the migration behavior of HUVECs under CoCl_2_-induced chemical hypoxia, which causes nuclear translocation of HIF-1α. Supplementation with CoCl_2_ at a concentration of more than 200 µM decreased the migration speed of HUVECs (Fig. S5(a)). The nuclear translocation of HIF-1α was significantly increased with the addition of 300 µM CoCl_2_ (Fig. S5(b)). Consequently, the migration speed was measured when HIF-1α was stabilized by 300 µM CoCl_2_ and PAK was inhibited by FRAX597. The migration speed of HUVECs was significantly decreased by the stabilization of HIF-1α, and it further decreased by PAK inhibition (Figure 6(a)). The nuclear translocation of HIF-1α was clearly observed even under the normoxic condition N (Figure 6(b)), and the ratio of the nuclear translocation of HIF-1α was equivalent to that under the most severe hypoxic condition, H0, without PAK inhibition (Figure 6(c)). Thus, we confirmed that hypoxia, similar to the most severe hypoxic condition H0, was chemically generated by CoCl_2_. The quantified ratio of the VE-cadherin area to the total cell area indicated that the stabilization of VE-cadherin was reduced by the addition of 300 µM CoCl_2_ (Figure 6(d)). However, VE-cadherin stabilization was recovered by PAK inhibition, showing the same level as the oxygen condition H0 without CoCl_2_. Based on these results, we conclude that PAK likely upregulates the HIF-1α-mediated hypoxic migratory response in HUVECs with VE-cadherin internalization.

## Discussion

The migration velocity of vascular endothelial cells is oxygen dependent; it tends to increase by hypoxic exposure, while it decreases under an extremely low oxygen tension of ≤1% O_2_. The increase in migration velocity under hypoxic conditions is partly due to the weakening of cell-cell adhesion by the internalization of VE-cadherin through the PAK pathway. Also, PAK upregulates HIF-1α, and the stabilization of HIF-1α upregulates or downregulates the migration velocity.

The migration speed of vascular endothelial cells was increased by hypoxic exposure, except under the extremely low oxygen condition H0, compared to the normoxic condition N, with the local maximum value under condition H1 (Figure 2). HIF-1α accumulated in vascular endothelial cells as the oxygen tension decreased, showing its highest nuclear translocation under the hypoxic condition H0 (Figure 3). In addition, the protein localization level of VE-cadherin in vascular endothelial cells was significantly decreased under the hypoxic condition H2 or below (Figure 4). Conversely, the inhibition of PAK in vascular endothelial cells by FRAX597 suppressed the internalization of VE-cadherin (Figure 4) and decreased the nuclear translocation of HIF-1α (Figure 5(b)). PAK inhibition decreased the migration speed of vascular endothelial cells under all oxygen conditions, except for the extremely low oxygen condition H0, and showed a remarkable decrease under condition H1 (Figure 5(a)). The decrease of the migration speed in vascular endothelial cells agrees with a previous study in which VE-cadherin was enhanced by forskolin [27]. However, under the extremely low oxygen condition H0, the migration speed was lower than that under the normoxic condition N (Figure 2). In addition, in contrast to the results under other conditions, PAK inhibition increased the migration speed of vascular endothelial cells to almost the same level as that under the normoxic condition N, though it suppressed the nuclear translocation of HIF-1α (Figure 5). HIF-1α has been reported to suppress oxidative phosphorylation in mitochondria in severe hypoxic conditions [36], and it is also known that intracellular ATP production is closely related to cellular dynamics [37,38]. Therefore, the mechanism of the cellular response under the oxygen condition H0 could be different from that under the other oxygen conditions. Several previous studies have also reported specific cellular responses in severe hypoxic conditions [39,40]. Further experiments with CoCl_2_, which maintained the high nuclear translocation of HIF-1α at the same level as in the oxygen condition H0, even with PAK inhibition, indicate specific variations of the migration speed of vascular endothelial cells (Figure 6). The migration speed of vascular endothelial cells became lower by the addition of CoCl_2_ (Figure 6(a)) and was further decreased by PAK inhibition to the same value as for condition H0, correlating with the VE-cadherin localization level (Figure 6(d)). DMSO, which was utilized as a solvent for FRAX597 and CoCl_2_, did not affect the migration speed, VE-cadherin localization level, or the nuclear translocation of HIF-1α (Fig. S4). Consequently, PAK is considered to upregulate HIF-1α, and the stabilization of HIF-1α upregulates or downregulates the migration velocity of vascular endothelial cells.

In our former study [27], a hypoxic condition of ~2% O_2_ decreased the area of VE-cadherin of HUVECs observed in microscopic images and increased collective cell migration. In this study, the oxygen concentrations at the center of the media channels under oxygen conditions H1 and H2 were 1.7% and 2.7%, respectively, and condition H1 was the closest to that applied in the former study. In fact, the migration speeds were similar between the present and former experiments. PAK is known to control the internalization of VE-cadherin [34], and PAK inhibition actually increased the protein localization level of the intercellular VE-cadherin. In addition, there is reportedly a feed-forward relationship between PAK and HIF-1α[31], and a decrease in the nuclear translocation of HIF-1α was observed by PAK inhibition. The CoCl_2_-induced chemical hypoxia caused the nuclear translocation of HIF-1α in vascular endothelial cells at the same level as the extremely low oxygen tension (Figure 6 and S5). Further investigations are necessary to elucidate the mechanisms by which HIF-1α accumulates and of the increase and decrease of migration velocity of vascular endothelial cells, which switched between oxygen conditions H0 and H1. Moreover, FRAX597 and CoCl_2_ were used for PAK inhibition and HIF-1α stabilization at concentrations that did not affect cell viability, but there remains a possibility that those chemicals secondarily affected signal transduction orthogonal to their intended purpose. Therefore, in future work we will conduct experiments using knockdown or knockout PAK, VE-cadherin, and HIF-1α cells. Furthermore, the hypoxic condition H6 caused no notable nuclear translocation of HIF-1α, but it still increased the migration speed of HUVECs. Investigating the HIF-1α-independent mechanism of hypoxic exposure will be a subject for future research.

## Supplementary Material

Supplemental Material
