# Supplementary material for: P21-activated kinase regulates oxygen-dependent migration of vascular endothelial cells in monolayers
Source: Cell Adh Migr. 2021 Sep 22;15(1):272–84. doi: 10.1080/19336918.2021.1978368 (PMC8475594; doi:10.1080/19336918.2021.1978368)
Supplement: Supplemental Material [file KCAM_A_1978368_SM5671.zip › supplementary/SupplementaryMaterials_210808.docx]

Fig. S1 Representative velocity vectors obtained by PIV analysis with sequential phase-contrast microscopic images between 30 min in the early stage (1–1.5 h), the middle stage (2.5–3 h), and the final stages (4.5–5 h) of the measurement period. The vectors colored based on the magnitude are displayed on the microscopic image. Scale bar = 100 µm.


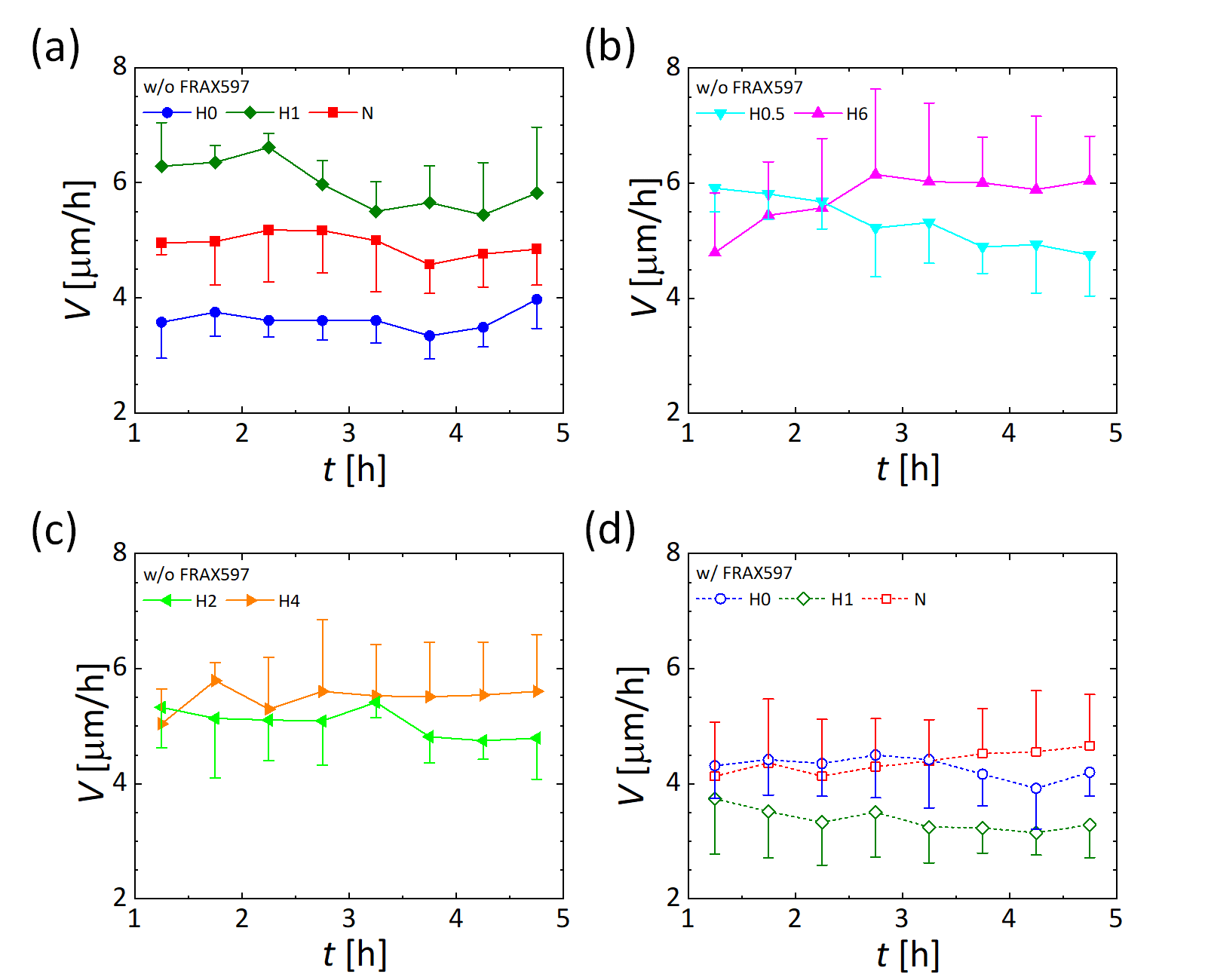


Fig. S2 Time variations of spatially averaged migration speed of HUVECs in the monolayer (a)–(c) without and (d) with the addition of the PAK inhibitor FRAX597.


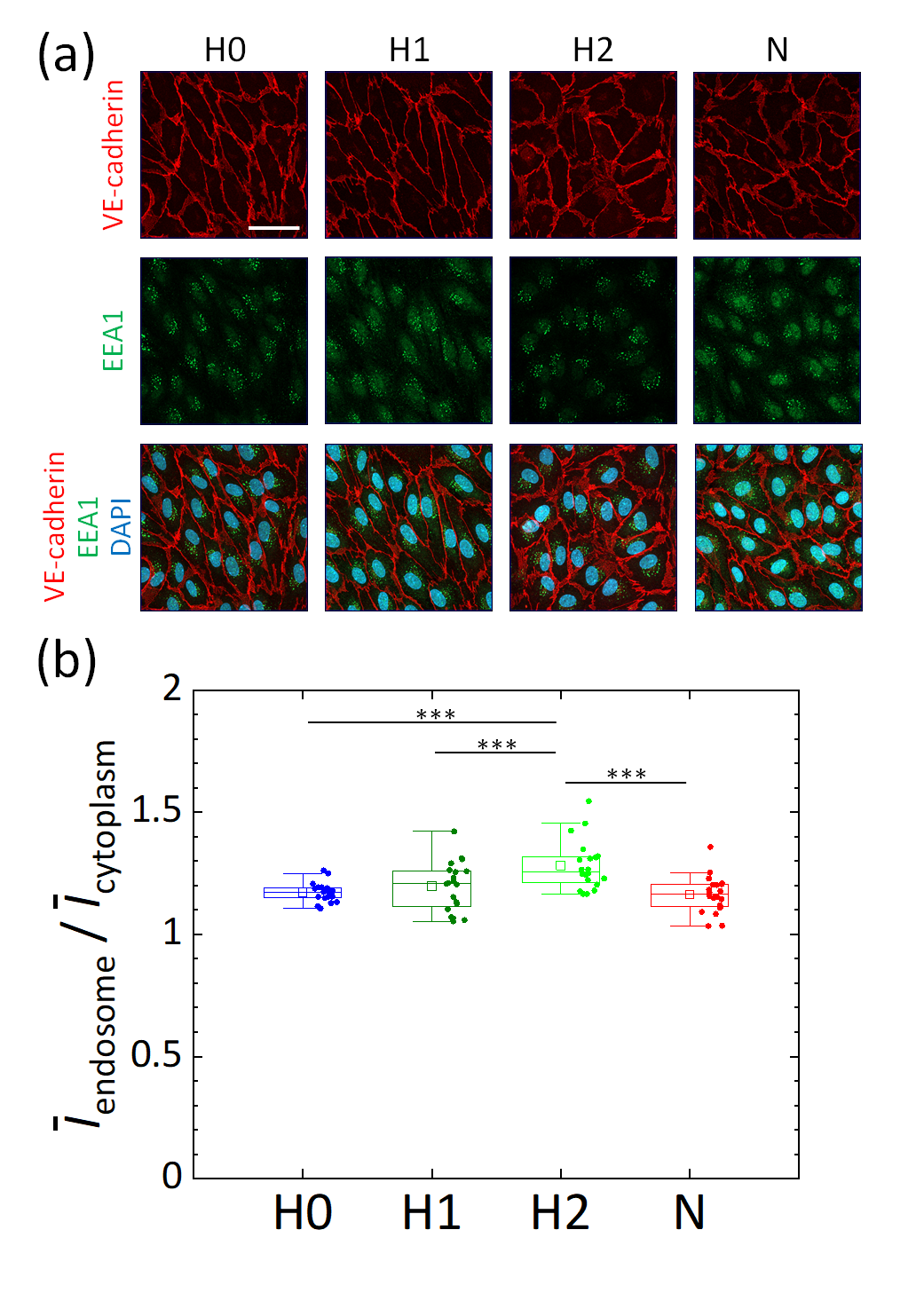


Fig. S3 VE-cadherin and EEA1 of HUVECs after 2 h of exposure to various oxygen conditions generated in the microfluidic device. (a) Representative images of maximum intensity projections of confocal microscopic images of HUVECs to the *xy*-plane. Scale bar = 50 µm. (b) Box-and-whisker plots of the average intensity of VE-cadherin colocalized with EEA1 relative to that in the cytoplasm, *Ī*_endosome_/*Ī*_cytoplasm_. The upper and lower extremes represent the maximum and minimum values, the box plot represents quartiles, and the band and open square inside each box show the median and the average value, respectively. The metric was quantified for 20 images from four devices under each condition, and the raw data are plotted with dots. Significant differences of the VE-cadherin area by oxygen concentration were assessed by one-way ANOVA followed by Tukey’s *post hoc* test for multiple comparisons. ****p* < 0.001.


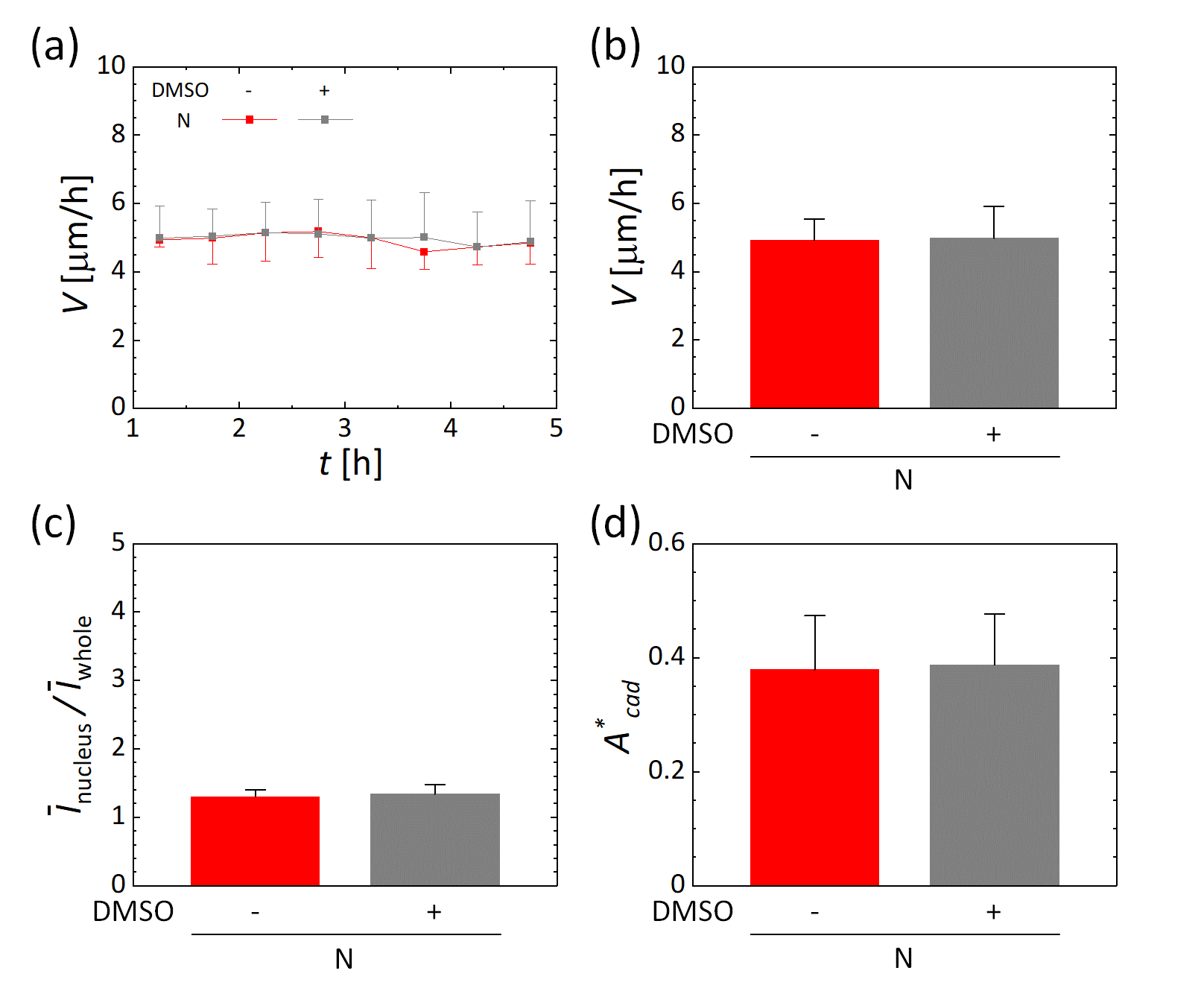


Fig. S4 Effects of DMSO added to the medium at a 1:1000 ratio. (a) Time variations of spatially averaged migration speed and (b) the spatiotemporally averaged migration speed of HUVECs in the monolayer. (c) Average intensity of HIF-1α in the nucleus relative to that in the whole image, *Ī*_nucleus_/*Ī*_whole_, and (d) ratio *A*^*^_cad_ of the VE-cadherin area to the total cell area. Error bars show standard deviation. No significant difference was detected between the two conditions by Welch’s t-test.


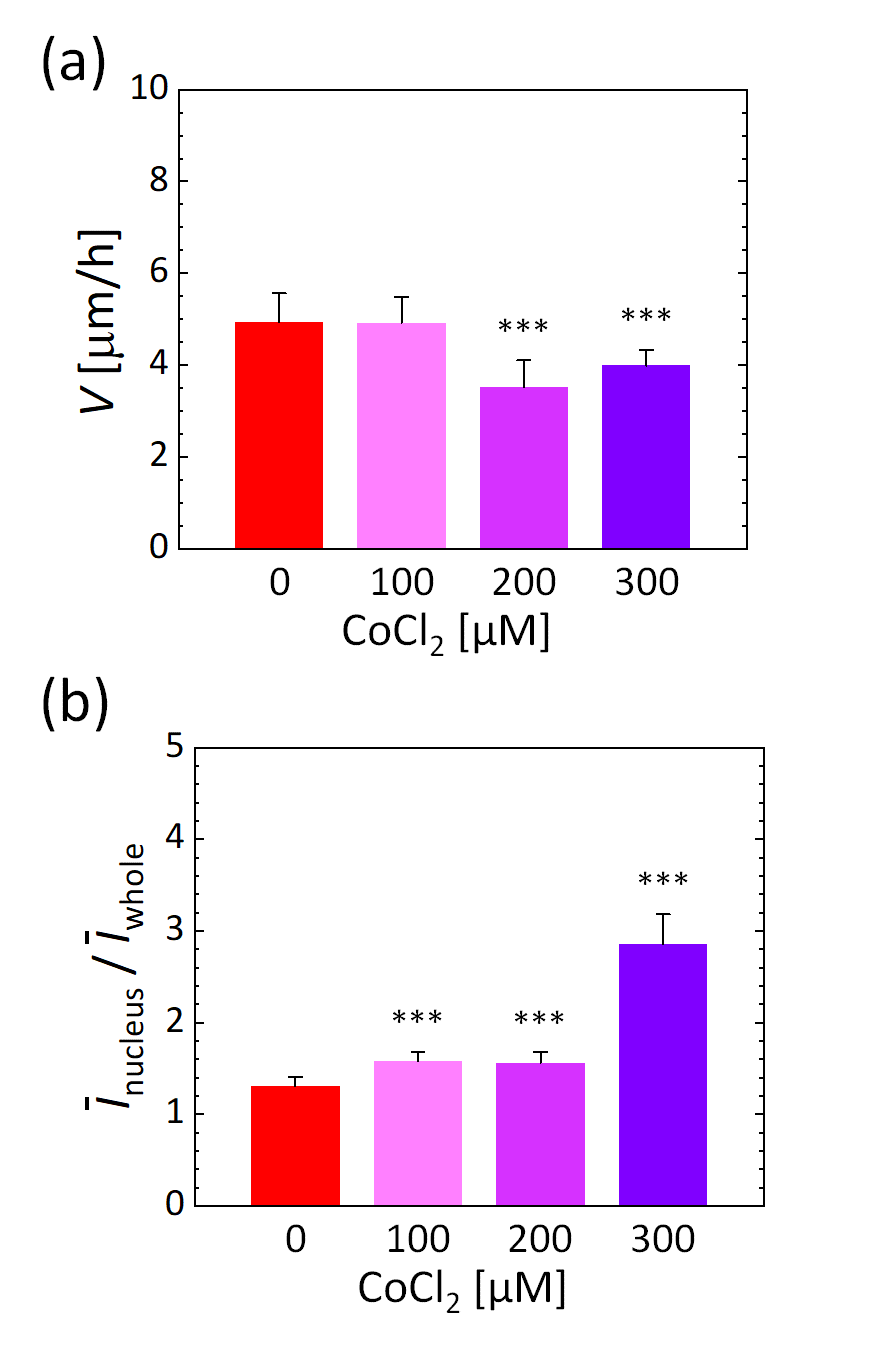


Fig. S5 Chemically-induced hypoxic conditions by CoCl_2_. (a) The spatiotemporally averaged migration speed and (b) average intensity of HIF-1α in the nucleus relative to that in the whole image, *Ī*_nucleus_/*Ī*_whole_. Error bars show standard deviation. Significant differences by CoCl_2_ concentration were assessed by one-way ANOVA followed by Tukey’s *post hoc* test for multiple comparisons. ****p* < 0.001 vs. control (without CoCl_2_).


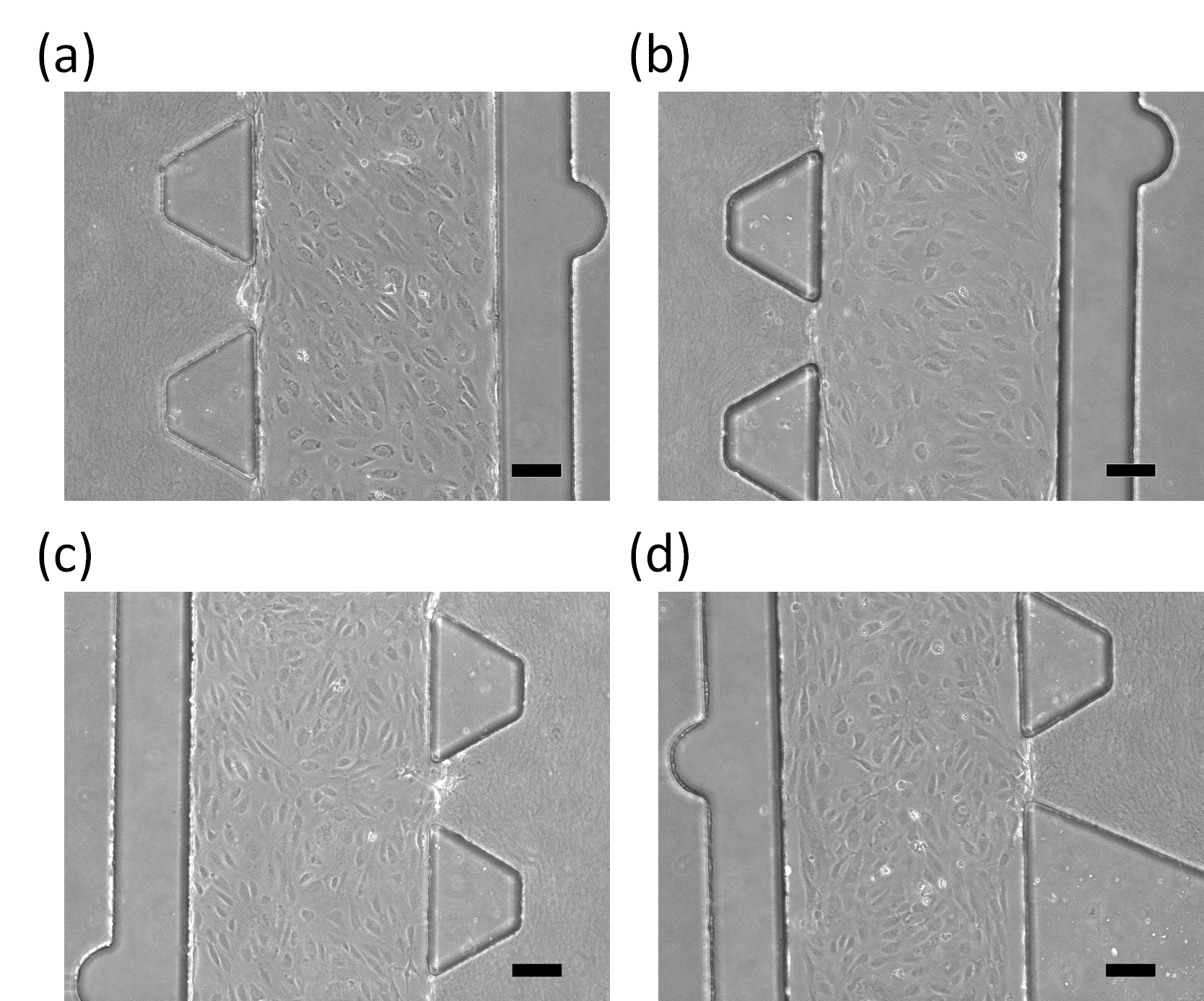


Movie S1 The collective cell migration of the HUVECs for 5 h at 3,600× speed under oxygen conditions (a) H0, (b) H1, (c) H2, or (d) N. Scale bar = 100 µm.
